# High prevalence of PI resistance in patients failing second-line ART in Vietnam

**DOI:** 10.1093/jac/dkv385

**Published:** 2015-12-11

**Authors:** Vu Phuong Thao, Vo Minh Quang, Jeremy N. Day, Nguyen Tran Chinh, Cecilia M. Shikuma, Jeremy Farrar, Nguyen Van Vinh Chau, Guy E. Thwaites, Sarah J. Dunstan, Thuy Le

**Affiliations:** 1 Wellcome Trust Major Overseas Programme, Oxford University Clinical Research Unit, Ho Chi Minh City, Vietnam; 2 Hospital for Tropical Diseases, Ho Chi Minh City, Vietnam; 3 Centre for Tropical Medicine and Global Health, Nuffield Department of Medicine, University of Oxford, Oxford, UK; 4 Hawaii Center for AIDS, University of Hawaii at Manoa, Honolulu, HI, USA; 5 The Peter Doherty Institute for Infection and Immunity, The University of Melbourne, Melbourne, Victoria, Australia

## Abstract

**Background:**

There are limited data from resource-limited settings on antiretroviral resistance mutations that develop in patients failing second-line PI ART.

**Methods:**

We performed a cross-sectional virological assessment of adults on second-line ART for ≥6 months between November 2006 and December 2011, followed by a prospective follow-up over 2 years of patients with virological failure (VF) at the Hospital for Tropical Diseases, Vietnam. VF was defined as HIV RNA concentrations ≥1000 copies/mL. Resistance mutations were identified by population sequencing of the *pol* gene and interpreted using the 2014 IAS-USA mutation list and the Stanford algorithm. Logistic regression modelling was performed to identify predictors of VF.

**Results:**

Two hundred and thirty-one patients were enrolled in the study. The median age was 32 years; 81.0% were male, 95.7% were on a lopinavir/ritonavir-containing regimen and 22 (9.5%) patients had VF. Of the patients with VF, 14 (64%) carried at least one major protease mutation [median: 2 (IQR: 1–3)]; 13 (59%) had multiple protease mutations conferring intermediate- to high-level resistance to lopinavir/ritonavir. Mutations conferring cross-resistance to etravirine, rilpivirine, tipranavir and darunavir were identified in 55%, 55%, 45% and 27% of patients, respectively. Higher viral load, adherence <95% and previous indinavir use were independent predictors of VF. The 2 year outcomes of the patients maintained on lopinavir/ritonavir included: death, 7 (35%); worsening virological/immunological control, 6 (30%); and virological re-suppression, 5 (25%). Two patients were switched to raltegravir and darunavir/ritonavir with good HIV control.

**Conclusions:**

High-prevalence PI resistance was associated with previous indinavir exposure. Darunavir plus an integrase inhibitor and lamivudine might be a promising third-line regimen in Vietnam.

## Introduction

The WHO endorses ritonavir-boosted PI (PIr)-based ART as an efficacious second-line treatment after failure of NNRTI-based first-line therapy in resource-limited settings.^1^ PIr-based therapy is highly potent in ART-naive patients participating in clinical trials^2–4^ and has a high efficacy as a second-line therapy in resource-limited settings.^5,6^ Nevertheless, ≤20% of patients in resource-rich and 27% of patients in resource-limited settings develop virological failure (VF) on PIr-based ART.^4,6,7^ PI resistance is rarely observed in patients failing PIr-based therapy in clinical trials^3,4,8,9^ and, similarly, is uncommon (range: 0%–7%) in PI-naive patients failing second-line therapy in sub-Saharan Africa.^10–14^ However, studies from Cambodia^15^ and India^16^ have reported PI-resistance-mutation prevalences of 40% and 70% in patients failing second-line ART, respectively. There are few data regarding the prevalence of and risk factors for PI resistance developed on second-line ART in Asia. Significant uncertainty exists regarding the risk factors for PI resistance in programmatic settings, the contribution of HIV-1 subtypes to mutation development and the clinical outcomes in patients with PI resistance on long-term second-line ART. HIV-1 subtype CRF01_AE accounts for 99% of HIV infections in Vietnam,^17–21^ which is among the Asian countries with the highest numbers of HIV infections.^22,23^ Of the 90 000 people on ART, 3% are on second-line therapy.^23^Because of its costs, viral load monitoring of HIV is not performed routinely. Therefore, data on virological outcome and drug resistance in patients on second-line therapy are lacking. To this end, we aimed to generate data on antiretroviral resistance profiles of HIV-1 CRF01_AE-infected patients with viraemia on second-line PI therapy at the largest HIV treatment centre in Vietnam. Our objectives were: (i) to identify the risk factors for resistance development; (ii) to describe the long-term clinical outcomes of patients with resistance maintained on a failing second-line regimen; and (iii) to investigate cross-resistance to second-generation NNRTIs and PIs to inform national policy on third-line therapy.

## Methods

### Study setting and design

The study was conducted at the Hospital for Tropical Diseases (HTD) in Ho Chi Minh City (HCMC). The HTD is the largest centre for HIV care in southern Vietnam, providing ART for more than 5000 patients according to the national ART programme. Until the de-centralization of care in 2011–12, the HTD had been the primary provider of second-line ART for patients living in the 17 southern provinces of Vietnam. First-line therapy was administered according to national and international guidelines and at the time of the study consisted of two NRTIs (lamivudine in combination with either zidovudine or stavudine) and one NNRTI—either nevirapine or efavirenz. Indinavir was generically and locally produced (STADA, Vietnam) during this time and was prescribed (without ritonavir boosting) in public and private settings for patients with treatment failure or intolerance on nevirapine before efavirenz became available in 2004.^24^ In 2011, tenofovir disoproxil fumarate replaced stavudine as a preferred NRTI backbone drug. Patients in the national programme were required to attend monthly appointments for clinical and adherence evaluation. CD4 cell count was performed every 6 months. HIV load testing was performed to confirm treatment failure when the WHO's defined clinical and/or immunological failure criteria were met.^25,26^ HIV genotyping was performed to diagnose antiretroviral resistance prior to therapy switch, and results were reported to treating clinicians. Second-line therapy included nelfinavir prior to 2006 and lopinavir/ritonavir thereafter, in combination with tenofovir disoproxil fumarate and/or zidovudine plus lamivudine.^27,28^

This study consisted of a cross-sectional survey of adult patients (age ≥15 years) who had been on second-line ART for at least 6 months and were on active care to identify those with VF and their drug resistance development, followed by a prospective follow-up over 2 years of patients with VF. VF was defined as at least two viral loads ≥1000 copies/mL measured 1–3 months apart after intensive adherence counselling. The patients who had been on second-line therapy for <6 months at the time of study assessment, who died or who switched therapy due to drug intolerance were excluded. The study was conducted between December 2011 and June 2014.

### Data collection

Clinical data, including demographic information, HIV risk factors, ART history, CD4 counts, HIV viral load, genotyping results at the time of therapy switch (if available), AIDS events and therapy adherence, were obtained both retrospectively and prospectively from patients' charts and from one-on-one interviews.

### ART adherence evaluation

Treating clinicians routinely assessed adherence according to the national guidelines at all clinic visits; adherence was recorded either as an estimated percentage of pills taken or as a qualitative assessment of ‘good’, ‘average’ or ‘poor’, corresponding to ≥95%, 80%–94% or <80% adherence, respectively.^26^ Additionally, for this study, adherence was evaluated over the 6 months preceding the time of study assessment using a simple self-reported visual analogue scale (VAS).^29^ For analysis, sub-optimal adherence was defined as having at least one adherence score of <95% by pill count or by the VAS and/or receiving at least one qualitative adherence assessment of ‘average’ or ‘poor’ over the preceding 6 months prior.

### HIV RNA measurement and antiretroviral resistance testing

At the time of enrolment, 5 mL of EDTA blood was collected for viral load measurement using the Abbott m2000rt Real Time HIV-1 assay (limit of detection of 150 copies/mL) (Abbott Laboratories, Abbott Park, IL, USA). Antiretroviral resistance testing was performed for patients with VF using an in-house population sequencing and sequence analysis protocol as previously described, with bidirectional coverage of the complete protease gene and reverse transcriptase codons 10–300.^18^ The sequences were analysed using SeqScape (Applied Biosystems). Nucleotide changes were determined by comparison with the consensus sequence pNL4-3 for HIV-1 subtype B (GenBank accession number M19921). Antiretroviral resistance mutations were identified based on the 2014 IAS-USA mutation list.^30^ The antiretroviral resistance profile of each patient was predicted using the Stanford resistance interpretation algorithm (http://hivdb.stanford.edu). The Rega HIV-1 subtyping tool was used to determine the HIV-1 subtype of each patient sample.^31^

### Statistical analyses of predictors of VF

The following pre-defined covariates were included in the logistic regression model: CD4 cell count and (log_10_-transformed) HIV RNA load at therapy switch, history of indinavir use, second-line therapy delay (defined as time in months from first detection of failure of first-line ART to time of second-line therapy initiation) and an overall measure of therapy adherence (<95% versus ≥95%). The chosen covariates were either established risk factors for ART outcome^5,32–36^ or were based on clinicians' observations (i.e. previous indinavir use). Both univariate and multivariable analyses were performed.

### Follow-up of patients with VF

The results of viral load and resistance testing were reported to the treating clinicians. Patients with VF then received intensive adherence counselling. As third-line therapy was not available through the national programme, these patients were continued on the current treatment according to national guidelines. The clinical and immunological outcomes of these patients over the following 24 months were evaluated. HIV RNA load was re-tested at month 24, and repeat genotype testing was performed if HIV RNA concentrations were ≥1000 copies/mL, to evaluate the evolution of resistance mutations in these patients.

### Ethics

The study was approved by the scientific and ethics committee of the HTD. All patients gave written consent prior to study enrolment.

## Results

### Study population and characteristics

**Figure 1.**
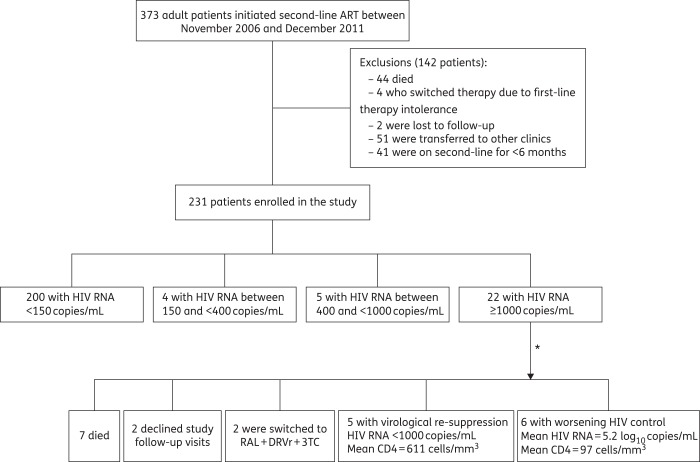
Flow chart of the study participants, virological outcome and follow-up of patients with VF maintained on the failing second-line ART. *24 months follow-up. DRVr, ritonavir-boosted darunavir; RAL, raltegravir; 3TC, lamivudine.

Figure 1 describes the study participants, virological outcome and follow-up of the patients with VF maintained on the failing second-line regimen. Of 373 patients who started second-line ART between November 2006 and December 2011, 44 (11.8%) had died, 2 had been lost to follow-up and 51 (13.7%) had been transferred to other provincial clinics by the time of the study. Forty-one (11.0%) patients who had been on second-line ART for <6 months and four patients who switched therapy due to first-line therapy intolerance were excluded. The remaining 231 patients were enrolled into the study. Table 1 shows the characteristics of the 231 patients. The median age was 32 years; 81% were men. The median CD4 cell count and HIV RNA concentration at the time of therapy switch were 44 cells/mm^3^ and 5.1 log_10_ copies/mL, respectively. The median time on second-line ART was 29 months (IQR: 16–43 months). Nelfinavir was the starting PI in 10 (4.3%) patients, but it was replaced by lopinavir/ritonavir within 12 months for all patients. A total of 36 (17.1%) patients had a history of treatment with indinavir, which was frequently prescribed at 800 mg twice daily or 400 mg three times daily. Sub-optimal adherence was identified in 12.1% patients.

**Table 1.** Characteristics of 231 patients on second-line ART in HCMC

Characteristic	
Male, *n* (%)	187 (81.0)
Age (years), median (IQR)	32 (28–36)
Previous history of injecting drug use, *n* (%); *N* = 230	93 (40.4)
CD4 count (cells/mm^3^), median (IQR); *N* = 227	44 (17–84)
HIV RNA concentration (log_10_ copies/mL), median (IQR); *N* = 215	5.1 (4.6–5.5)
Previous indinavir use, *n* (%); *N* = 211	36 (17.1)
Time on second-line therapy (months), median (IQR)	29 (16–43)
Second-line regimens, *n* (%)	
initial regimens	
TDF/3TC/LPVr	112 (48.5)
TDF/3TC/LPVr + AZT	82 (35.5)
LPVr + other NRTIs^a^	27 (11.7)
NFV + other NRTIs^a^	10 (4.3)
regimens at time of study assessment	
TDF/3TC/LPVr	128 (55.4)
TDF/3TC/LPVr + AZT	88 (38.1)
LPVr + other NRTIs^a^	15 (6.5)
Adherence, *n* (%)	
≥95%	203 (87.9)
<95%	28 (12.1)

TDF, tenofovir disoproxil fumarate; 3TC, lamivudine; LPVr, lopinavir/ritonavir; AZT, zidovudine; NFV, nelfinavir.

^a^Other NRTIs include two or three of the following drugs: abacavir, didanosine, zidovudine, lamivudine, stavudine or tenofovir disoproxil fumarate.

### Antiretroviral resistance mutations detected in patients prior to second-line therapy switch

**Figure 2.**
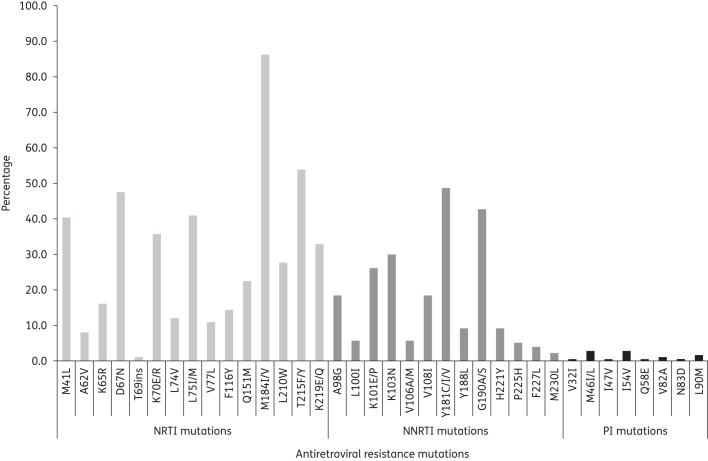
Prevalence of antiretroviral resistance mutations in 173 patients at the time of switch to second-line therapy in HCMC.

Because of cost constraints, HIV genotyping was performed only for 173 of 231 (74.9%) patients prior to therapy switch. Figure 2 shows the mutations and prevalences detected in these patients. Mutations conferring high-level resistance to NRTIs were detected in 168 of 173 (97.1%) patients, and to NNRTIs in 163 of 173 (94.2%) patients. High-level resistance to PIs was detected in 4 of 173 (2.3%) patients. Resistance mutations to both NRTIs and NNRTIs were present in 161 of 173 (93.1%) patients and to all three drug classes in 6 of 173 (3.5%) patients. The most common NRTI resistance mutations were M184I/V (86.1%), thymidine analogue mutations M41L, D67N, K70E/R, T215F/Y and K219E/Q (33%–57%), Q151M (22.5%) and K65R (16.2%). One hundred and forty-two (82.1%) patients harboured multiple thymidine analogue mutations and multiple NRTI resistance mutations (Q151M complex). Two patients had a T69 insertion mutation. The most common NNRTI resistance mutations were Y181C/I/V (48.6%), G190A/S (42.8%) and K103N (30.1%). At least three major NNRTI resistance mutations were present in 55 of 173 (31.8%) patients. Eight patients carried at least one major PI resistance mutation. The most common protease mutations were M46I/L (2.9%), L90M (1.7%) and V82A (1.2%).

### Predicted resistance to second-line ART regimen

The predicted susceptibilities to the national second-line regimens containing tenofovir disoproxil fumarate, lamivudine and lopinavir/ritonavir were evaluated for the 173 patients who had genotype results using the Stanford HIV Drug Resistance algorithm. Intermediate- to high-level resistance to tenofovir disoproxil fumarate was present in 120 of 173 (69.4%), to lamivudine in 165 of 173 (95.4%) and to lopinavir/ritonavir in 2 of 173 (1.2%).

The numbers of patients predicted to receive one, two and three fully active drugs were 138 of 173 (79.8%), 25 of 173 (14.5%) and 4 of 173 (2.3%), respectively.

### Virological outcome

The virological outcomes of the 231 patients are shown in Figure 1. Twenty-two (9.5%) patients had confirmed VF with a median HIV RNA concentration of 4.75 log_10_ copies/mL (IQR: 3.92–5.01 log_10_ copies/mL). Five of 231 (2.2%) patients had HIV RNA concentrations between 400 and <1000 copies/mL, 4 (1.7%) patients had HIV RNA concentrations between 150 and <400 copies/mL and the remaining 200 (86.6%) patients had undetectable viral loads.

### HIV subtypes, antiretroviral resistance mutations and predicted susceptibility of the 22 patients with VF

Of the 22 patients with VF, 21 (95%) were infected with HIV-1 subtype CRF01_AE; a single patient was infected with HIV-1 CRF01_AE/B recombinant. Table 2 shows the mutation profiles of the 22 patients prior to therapy switch and at VF. The majority of the NRTI and NNRTI resistance mutations detected prior to therapy switch remained detectable at therapy failure, with the NNRTI resistance mutations persisting up to 45 months off NNRTI therapy. Major PI resistance mutations developed in 14 (64%) patients; within this subgroup of patients, the median number of PI resistance mutations was 2 (IQR: 1–3 mutations). The most common PI resistance mutations were V82A/F (64%), M46I/L (57%), I84V (29%) and L76V (21%). Five patients had only one PI resistance mutation; the remaining nine had multiple PI resistance mutations. Minor or accessory PI resistance mutations developed in five patients. Three patients did not have any PI resistance mutations.

**Table 2.** Antiretroviral history, drug resistance profile and 2 year outcomes of 22 patients with VF on second-line ART in HCMC

Patient	Time on second- line ART (months)	At time of therapy switch		Prior PI use	Mutations at time of therapy switch			At time of VF		Mutations at time of VF			Two year outcomes
		CD4 count (cells/mm^3^)	viral load (copies/mL)		NRTIs	NNRTIs	PIs	CD4 count (cells/mm^3^)	viral load (copies/mL)	NRTIs	NNRTIs	PIs	
1	15	79	4 720 000	no	**L74V, M184I**	**K101E, K103N, G190A, M230L**		191	289 000	**D67N, K70R, L74V, M184I, K219Q**	**K101E, K103N,** **E138G, G190A,** **M230L**	L10I, **V82A**	virological re-suppression
2	18	2	7 590 000	NA	NA	NA	NA	2	1 630 000	**M184V**	**K103N, V108I, Y181C**		death
3	19	13	170 000	IDV	NA	NA	NA	50	402 194	T215S		G16E, K20I, M36I, **M46L,** I54V, H69K, **V82A,** L89M	death
4	18	74	118 000	IDV	**M41L, D67N, K70R,** V75M, **M184V, T215F, K219Q**	**K101P, K103N**		303	1574	**M41L, D67N, K70R,** V75M, **M184V, T215F, K219Q**	**K101P, K103N**	L10I, G16E, K20I, M36I, H69K, L89M	transferred to other clinic
5	18	45	435 000	IDV	**A62V, K65N,** T69S, V75M, **F77L, Q151M, M184V**	V106I, **Y181C, Y188L, H221Y**	I54V, **N83D,** I84R	152	1184	V75M, **M184V, T215F**	V106I, **Y181C, Y188L, H221Y**	L10I, K20I, M36I, **M46L,** F53L, I54V, H69K, **V82A,** L89I	worsening virological/immunological control
6	29	5	365 000	no	**K65R, Q151M**	**Y181C, G190A**	L33F, I84L	143	5490	**K65R, Q151M**	**Y181C, G190A**	K20R, L33F, M36I, **M46I,** I62V, H69K, **L76V, I84V,** L89M	worsening virological/immunological control
7	6	6	190 000	IDV	**A62V, D67N,** T69P, **V75I, F77L, F116Y, Q151M, M184I,** T215S, **K219Q**	**K101E, Y181C, G190A**	L10V	293	3910	**D67N, V75I, F77L, F116Y, Q151M, M184I, K219Q**	**K101E, Y181C, G190A**	L10V, G16E, M36I, H69K, **V82A,** L89M	virological re-suppression
8	31	40	184 000	no	**M41L, D67N,** T69N, **K70R,** L74I, **M184V, T215F, K219Q**	**V108I,** **G190A**	L10IV	546	1520	**M41L, D67N, K70R,** L74I, **M184V, T215F, K219Q**	**G190A**	L10V, G16E, L33F, M36I, I54V, **V82A,** L89I	virological re-suppression
9	47	8	253 000	IDV	**M41L, D67N,** T69N, **K70R,** L74I, **M184V, T215F, K219Q**	A98G, **K103N, G190A**		171	37 379	**M41L, D67N, K70R, M184V, T215F, K219Q**	**K103N, G190A**	L10V, K20I, L33F, M36L, **M46I,** **I47V,** I54V, H69K, **T74P,** **V82F,** L89M	worsening virological/immunological control
10	8	21	752 000	NA	NA	NA	NA	152	16 582	**K65R,** V75M	V179F, **Y181C, H221Y**	M36I, H69K, L89M	virological re-suppression
11	45	21	867 000	no	**M41L,** E44AD, **D67N, L74V,** V75M, V118I, **M184V, L210W, T215Y,** K219N	A98G, **L100I, K101P, G190A**		121	96 147	**M41L, D67N,** V75M, **M184V, L210W**	A98G, **G190A**	M36I, H69K, V82I, L89M	death
12	43	1	132 000	no	**D67N, K70R, M184V, L210W, T215F,** K219W	**K103N, V108I, Y181C, G190A**		11	22 600	**M184V**			death
13	50	113	38 238	IDV	**M41L, D67N,** T69P, **K70R, M184V, L210W, T215F, K219E**			57	319 798	**M41L, D67N, K70R, M184V, L210W, K219QE**		L10V, G16E, K20V, L33F, M36I, **I47V,** I54V, H69K, A71V, **I84V,** L89M	worsening virological/immunological control
14	12	25	693 000	no	NA	NA	NA	48	875 664			L10I, K20R, M36I, H69K, L89M	virological re-suppression
15	29	211	189 000	IDV	T69N, V75M		**V32I, M46I, Q58E**	253	64 262	**K70R,** V75M, **M184V, K219E**	V90I	L10I, G16E, K20I, M36I, **M46I,** I54A, **Q58E,** H69K, K70R, **V82A,** L89I	worsening virological/immunological control
16	47	44	139 000	IDV	**M41L,** T69N, V75M, **F77L, F116Y, Q151M, M184V, T215Y**	A98G, **L100I, K103N**		154	34 900	**M41L,** V75M, **F77L, M184V, T215Y**	A98G	L10I, G48A, I54V, A71V, **V82A**	RAL + DRVr + 3TC
17	43	64	174 550	IDV	**M41L, D67N, K70R, L74V, M184V, T215F, K219Q**	**Y181C, G190S**	M36I	67	47 500	**M41L, D67N, K70R, L74V, M184V, T215F, K219Q**	A98G, **Y181C, G190S**	L10I, L33F, **M46I, I54M,** A71V, G73S, **I84V**	death
18	23	41	377 000	no	**K65R,** V75M	**K103N**		6	67 934			M36I, H69K, V82I, L89M	worsening virological/immunological control
19	17	40	590 000	no	**D67N,** T69N, **K70R,** L74I, V75M, **M184V, T215F, K219E**	**K101P, Y181C, G190S**		352	3776	**D67N, K70R,** V75M, **M184V, T215F, K219E**	K101Q, **Y181C, G190A**	K20R, M36I, **M46I,** L63P, H69K, A71V, **L76V,** **I84V,** L89M	transferred to other clinic
20	30	3	948 909	NA	NA	NA	NA	98	103 000	**K70R, T215F, K219E**	A98G, **K101E, Y181C, Y188L, G190A**	L10I, I54V, **N83D**	death
21	45	113	2 470 000	IDV	**M41L, D67N,** V75M, V118I, **M184V, L210W, T215F,** K219W	**K101P, V108I, G190A**	L10F	173	79 800	**M41L, D67N,** V75M, **M184V, T215Y**	A98G	L10F, **M46L,** I54V, **L76V,** **V82A,** L89V	RAL + DRVr + 3TC
22	26	14	81 422	IDV	NA	NA	NA	28	83 300	**K65R**	**K101E, Y181C, G190A**		death

IDV, indinavir; RAL, raltegravir; DRVr, darunavir/ritonavir; 3TC, lamivudine; NA, not applicable (as data are unknown).

Bold: major drug resistance mutations according to the IAS-USA 2014.

**Figure 3.**
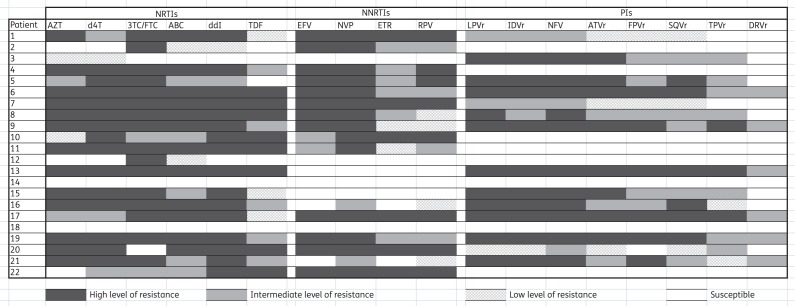
Predicted antiretroviral susceptibility among 22 patients experiencing VF on second-line ART in HCMC using the Stanford algorithm. AZT, zidovudine; d4T, stavudine; 3TC, lamivudine; FTC, emtricitabine; ABC, abacavir; ddI, didanosine; TDF, tenofovir disoproxil fumarate; EFV, efavirenz; NVP, nevirapine; ETR, etravirine; RPV, rilpivirine; LPV, lopinavir; IDV, indinavir; NFV, nelfinavir; ATV, atazanavir; FPV, fosamprenavir; SQV, saquinavir; TPV, tipranavir; DRV, darunavir; r, ritonavir-boosted.

Figure 3 shows the predicted resistance profiles of the 22 patients based on their individual genotype profiles. Mutations conferring intermediate- to high-level resistance to the second-line drugs tenofovir disoproxil fumarate, lamivudine and lopinavir/ritonavir were detected in 13 (59%), 18 (82%) and 13 (59%) patients, respectively. Cross-resistance to the second-generation NNRTIs etravirine and rilpivirine was intermediate to high level, and cross-resistance to both was present in 12 (55%) patients. Cross-resistance to the second-generation PIs tipranavir and darunavir was present in 10 (45%) and 6 (27%) patients, respectively. Cross-resistance to darunavir was present only at an intermediate level.

### Predictors of second-line virological outcome

Table 3 lists the data for the five covariates entered into the logistic regression model and the results of the univariate and multivariate analyses. The most frequently missing covariates were history of indinavir use (8.7% missing) and viral load (6.9% missing); other covariates were missing in ≤2% of patients. Higher viral load, sub-optimal adherence and previous indinavir use predicted VF in both univariate and multivariate analyses [multivariate ORs: 2.7 (95% CI: 1.1–7.4), *P* = 0.039; 7.8 (95% CI: 2.1–31.0), *P* = 0.002; and 12.8 (95% CI: 3.7–49.8), *P* < 0.001, respectively]. Multivariate analysis shown in Table 3 was based on an analysis excluding missing data.

**Table 3.** Factors associated with VF in 231 patients on second-line ART in HCMC

Covariate	Patients without VF (*N* = 209)	Patients with VF (*N* = 22)	Univariate effect		Multivariate effect	
			OR (95% CI)	*P*	OR (95% CI)	*P*
CD4 count^a^ (by −50 cells/mm^3^)	47 (17–88); *N* = 205^b^	33 (9–59)	1.39 (0.92–2.38)	0.184	1.52 (0.84–3.45)	0.248
HIV RNA concentration^a^ (by +log_10_ copies/mL)	5.1 (4.6–5.5); *N* = 194^b^	5.6 (5–5.9); *N* = 21^b^	**3.14 (1.56–6.69)**	**0.002**	**2.70 (1.08–7.35)**	**0.039**
Time on failing first-line ART (months)	9 (5–15); *N* = 206^b^	9 (3–21)	1.01 (0.97–1.05)	0.537	1.01 (0.95–1.07)	0.786
Adherence <95% (yes)	21 (10%)	7 (32%)	**4.18 (1.46–11.16)**	**0.005**	**7.81 (2.06–31.00)**	**0.002**
Prior indinavir use (yes)	27 (14%); *N* = 192^b^	9 (47%); *N* = 19^b^	**5.50 (2.02–14.91)**	**<0.001**	**12.80 (3.69–49.80)**	**<0.001**

Data are presented as absolute numbers (%) for categorical variables and median (IQR) for continuous variables.

Bold: results with statistical significance.

^a^At time of therapy switch.

^b^Number of patients with complete data on a covariate.

We performed *ad hoc* univariate and multivariate analyses of factors associated with the development of PI resistance. The three covariates identified to be independent predictors of VF were entered into the logistic regression model. Higher viral load and previous indinavir exposure remained independent predictors of PI resistance in both univariate [ORs: 2.7 (95% CI: 1.1–6.5), *P* = 0.03; and 9.7 (95% CI: 3.0–34.2), *P* < 0.001, respectively] and multivariate [ORs: 4.3 (95% CI: 1.5–14.8), *P* = 0.01; and 13.6 (95% CI: 3.6–61.3), *P* < 0.001, respectively] analyses. Adherence did not predict PI resistance in the univariate or multivariate analyses.

### Two year follow-up of patients with VF maintained on the failing second-line therapy

The clinical outcomes of the 22 patients with VF are shown in Figure 1 and are also reported along with their treatment history and resistance profiles in Table 2. Seven patients died after a median duration of 8 months (IQR: 8–16 months) from the time of study enrolment: four of tuberculosis and three of severe wasting syndrome. Of the 15 patients who remained alive, two patients were transferred to provincial clinics and declined study follow-up visits. Two patients were switched to raltegravir and darunavir/ritonavir (purchased privately from Thailand for $600 US/month) and remained on lamivudine. The remaining 11 patients were maintained on the lopinavir/ritonavir-based regimen. Viral load testing was performed at 24 month in the 13 patients in active follow-up. Virological re-suppression was achieved in the two patients who switched to raltegravir and darunavir/ritonavir and in five patients maintained on lopinavir/ritonavir. The CD4 cell counts of the five patients with virological re-suppression on lopinavir/ritonavir increased to a mean of 611 cells/mm^3^ (range: 384–942 cells/mm^3^). Of these five patients, two had no major PI resistance mutations and three had only one major mutation—V82A—at VF. The remaining six patients had persistent viral replication (mean HIV RNA concentration: 5.2 log_10_ copies/mL; range: 4.79–5.70 log_10_ copies/mL). Table 4 shows the evolution of antiretroviral resistance mutations of these six patients. Patient 18 had no major drug resistance mutations, and the other five had multiple major PI resistance mutations at VF and continued to accumulate NRTI resistance mutations (in five patients) and PI resistance mutations (in two patients). All six patients had worsening immunological control (mean CD4 count: 97 cells/mm^3^, range: 0–177 cells/mm^3^); however, there were no AIDS events over the 24 months of follow-up.

**Table 4.** Evolution of resistance mutations in six patients with worsening HIV control who were maintained on a failing second-line regimen

Patient	Time on second-line ART (months)	At time of VF		Mutations at time of VF			At 2 year follow-up		Mutations at 2 year follow-up		
		CD4 count (cells/mm^3^)	viral load (copies/mL)	NRTIs	NNRTIs	PIs	CD4 count (cells/mm^3^)	viral load (copies/mL)	NRTIs	NNRTIs	PIs
5	18	152	1184	V75M, **M184V, T215F**	V106I, **Y181C, Y188L, H221Y**	L10I, K20I, M36I, **M46L,** F53L, I54V, H69K, **V82A,** L89I	77	93 500	**A62V, K65N,** V75M, **F77L, Q151M, M184V**	V106I, **Y181C, Y188L, H221Y**	L10F, K20I, M36I, **M46L,** F53L, I54V, H69K, **V82A,** L89I
6	29	143	5490	**K65R, Q151M**	**Y181C, G190A**	K20R, L33F, M36I, **M46I,** I62V, H69K, **L76V, I84V,** L89M	177	164 000	**K65R,** **D67N,** T69d, **Q151M, K219E**	**Y181C, G190A**	L10F, K20R, L33F, M36I, **M46I,** I62V, H69K, **L76V, V82A,** T74S, **I84V,** L89M
9	47	171	37 379	**M41L, D67N, K70R, M184V, T215F, K219Q**	**K103N, G190A**	L10V, K20I, L33F, M36L, **M46I,** **I47V,** I54V, H69K, **T74P,** **V82F,** L89M	159	61 100	**M41L, D67N,** T69N, **K70R,** V75M, **M184V, L210W, T215F, K219Q**	V106I, **G190A**	L10V, K20I, L33F, M36L, **M46I, I47V,** I54V, H69K, **T74P, V82F,** L89M
13	50	57	319 798	**M41L, D67N, K70R, M184V, L210W, K219QE**		L10V, G16E, K20V, L33F, M36I, **I47V,** I54V, H69K, A71V, **I84V,** L89M	10	498 000	**M41L, D67N, K70R, M184V, L210W, T215Y,** K219D		L10V, G16E, K20V, L33F, M36I, **M46I, I47V,** I54V, H69K, A71V, G73T, L76M, **I84V,** L89T
15	29	253	64 262	**K70R,** V75M, **M184V, K219E**	V90I	L10I, G16E, K20I, M36I, **M46I,** I54A, **Q58E,** H69K, K70R, **V82A,** L89I	159	97 100	D67H, T69G, **K70R,** V75M, **M184V,** T215I, **K219E**	V90IV	L10I, G16E, K20I, L33F, M36I, **M46I,** I54A, **Q58E,** H69K, K70R, **V82A,** L89I
18	23	6	67 934			M36I, H69K, V82I, L89M	0	279 000		V106I	M36I, H69K, V82I, L89M

Bold: major drug resistance mutations.

## Discussion

We report the antiretroviral resistance profiles of patients failing second-line PI-based therapy in Vietnam. The major finding was that amongst the patients experiencing VF, 64% harboured at least one major PI resistance mutation and 60% had mutations that conferred intermediate- to high-level resistance to lopinavir/ritonavir. This level of PI resistance is significantly higher than has been previously reported in either resource-rich or resource-poor settings.^3,4,8–15,37^ Ritonavir-boosted PIs are known to have a high genetic barrier to resistance.^8,38^ The minimum plasma concentrations of ritonavir-boosted PIs far exceed the levels required to inhibit WT virus replication,^39,40^ making PIs a durable class of antiretroviral drug to be used across different patient populations. A high prevalence of PI resistance has been reported in four studies, two of which studied populations from Asia, specifically, from Cambodia (*N* = 71, 40%)^15^ and India (*N* = 45, 73%).^16^ The other two study populations were from West Africa: Mali (*N* = 93, 25%)^37^ and Nigeria (*N* = 61, 62%).^41^ Except for the study from India, where indinavir/ritonavir and atazanavir/ritonavir were commonly used, the studies in these other countries used lopinavir/ritonavir for second-line therapy. Previous exposure to generically produced un-boosted indinavir and nelfinavir was implicated in the observed high prevalence of PI resistance mutations in the reports from Asia and Nigeria, although formal analyses were lacking. Our study is the first to systematically link previous PI exposure to VF and PI resistance.

Indinavir was generically produced in Vietnam during the early 2000s. The correct dosing was 800 mg three times daily; however, because of the high rate of side effects, many Vietnamese clinicians prescribed it at 400 mg three times daily or 800 mg twice daily. A combination of high pill burden, short half-life, food restriction, high rate of side effects and inadequate dosing likely led to inadequate plasma drug concentrations and increased the risk of PI resistance in patients. Low plasma indinavir concentration has been shown to increase the risk of developing PI resistance mutations in patients experiencing early VF.^42^ Further, the most common PI resistance mutations detected in our cohort—M46I/L, I54V, V82A and L90M—were shown to be the first mutations to be sequentially selected by indinavir therapy.^43^ Cheap generically made indinavir, nelfinavir and saquinavir were available in India, China and south-east Asian countries during the same time.^15,44^ This availability likely explains the higher prevalence of PI resistance reported in the studies from Cambodia and India and suggests that the scope of PI exposure and resistance in Asia might be larger than is currently appreciated. Another reason for the high level of PI resistance observed in our study as well as these other studies is the lack of viral load monitoring, which leads to late detection of VF and accumulation of PI resistance mutations. Better understanding of the extent and determinants of PI resistance in developing countries is needed.

Among the next-generation NNRTIs and PIs potentially available as third-line drugs, there was evidence of probable intermediate or high levels of cross-resistance to etravirine, rilpivirine and tipranavir in ∼50% of patients. Cross-resistance to darunavir was less frequent (27%) and was observed only at the intermediate level. These prevalences are noticeably higher than those found in studies in similar settings.^15,16,37,41^ One reason for the observed high-level etravirine cross-resistance is programmatic. The lack of virological monitoring led to prolonged periods of undetected VF in the presence of the low-genetic-barrier drugs nevirapine and efavirenz and accumulation of resistance mutations. This effect was shown by the extensive NNRTI resistance mutations in our cohort (94% of patients with ≥1 and 32% with ≥3 major NNRTI resistance mutations). NNRTI resistance mutations have been shown to persist up to 45 months after the discontinuation of NNRTI therapy. This finding is due to the low fitness costs of these mutations on viral replication, thus explaining the slow reversion of these mutant viruses to WT in the absence of drug pressure.^45,46^ The presence of ≥3 IAS-USA-defined NNRTI resistance mutations has been associated with decreased virological response to etravirine in the DUET trials.^47,48^

Another reason for high etravirine cross-resistance is the inherent genetic variability of the HIV-1 subtype CRF01_AE in south-east Asia. Etravirine was designed to work against HIV containing the NNRTI signature mutation—K103N—which is highly prevalent in HIV-1 subtype B.^49^ However the most frequent NNRTI resistance mutations selected in subtype CRF01_AE virus by nevirapine and efavirenz exposure are Y181C and G190A/S, rather than K103N.^50,51^ In the DUET trials, the presence at baseline of these substitutions was associated with impaired virological response to etravirine.^47,48^ High prevalence of cross-resistance to etravirine (60%) has been reported in several studies of CRF01_AE-infected patients in Thailand for whom first-line NNRTI-based therapy was failing.^52–54^ As efficacy data of etravirine use in south-east Asia are lacking, phenotypic assays investigating the *in vitro* susceptibilities of these clinical isolates would be helpful. Until then, etravirine and rilpivirine should probably be avoided as third-line drugs for patients infected with subtype CRF01_AE in south-east Asia. Our data do not support the 2010 and 2013 WHO recommendations to use etravirine in a third-line ART regimen in resource-limited settings.^1,25^ The phenotypic susceptibility of tipranavir is not as well predicted as that of darunavir by most genotypic interpretation algorithms, in particular for non-B subtypes.^55^ However, based on our predicted cross-resistance data, darunavir/ritonavir plus a brand-new class of antiretroviral drug, such as integrase strand transfer inhibitors (INSTIs), combined with lamivudine is a reasonable third-line option for Vietnam. As the need for third-line therapy is imminent in the developing world, clinical trials evaluating cost-effective third-line treatment strategies and regimens are needed.

Among the 20 patients who were maintained on the failing lopinavir/ritonavir regimen, death or worse virological/immunological control occurred for 13 patients, with accumulation of resistance mutations occurring in those who remained alive at 24 months. This finding is consistent with that of a study from Nigeria showing accumulation of PI resistance mutations in patients maintained on failing second-line therapy.^41^ However, virological re-suppression and good immune response were achieved in five patients; these patients either had no or only one major PI resistance mutation at VF detection. A strategy combining adherence intervention and close monitoring of patients failing second-line therapy before switching to third-line therapy would be cost saving yet effective in resource-poor settings.

Our study has limitations. The study captured VF at one point in time and only in patients who were in active follow-up. The unavoidable exclusion of the 12% who had died and the 14% who had been transferred to their respective resident provinces reduces the power of our observations. Further, PI resistance might be underestimated due to the lack of data from those who had died. Nevertheless, the study site is the largest centre for second-line therapy in Vietnam. The highly uniform HIV care system along with standardized ART regimens in the national programme allow for reasonable generalizability of our findings. We did not sequence the integrase gene in this cohort, as INSTIs are not yet available in Vietnam.

In conclusion, we identified a significantly higher prevalence of PI resistance in patients failing second-line therapy in Vietnam, which was associated with previous indinavir exposure. The widespread availability of generically made PIs in Asia suggests that the scope of PI resistance might be underestimated in this region. Our data emphasize the need for viral load monitoring to limit the accumulation of NRTI and NNRTI resistance mutations, thus improving second-line treatment outcome and preserving the limited third-line therapy options. Significant cross-resistance to etravirine is common in subtype CRF01_AE-infected patients failing NNRTI therapy, suggesting that etravirine should be avoided as a third-line therapy drug. Research on cost-effective strategies and timing of third-line therapy switch are now needed.

## Funding

This work was supported by the Wellcome Trust.

## Transparency declarations

None to declare.

### Author contributions

Study concept and design: T. L., V. M. Q., C. S., S. D. and J. F. Obtaining funding: T. L., S. D., J. D. and J. F. Acquisition of data: V. P. T., V. M. Q., N. T. C., N. V. V. C. and T. L. Analysis and interpretation of the data: V. P. T. and T. L. Drafting the manuscript: V. P. T. and T. L. Critical revision of the manuscript for important intellectual content: V. M. Q., N. T. C., C. S., J. F., J. D., N. V. V. C., S. D. and G. T. All authors contributed to and approved the final manuscript.
